# Sarcopenia and adverse health‐related outcomes: An umbrella review of meta‐analyses of observational studies

**DOI:** 10.1002/cam4.3428

**Published:** 2020-09-13

**Authors:** Lin Xia, Rui Zhao, Qianyi Wan, Yutao Wu, Yong Zhou, Yong Wang, Yaping Cui, Xiaoding Shen, Xiaoting Wu

**Affiliations:** ^1^ Department of Gastrointestinal Surgery West China Hospital Sichuan University Chengdu China; ^2^ Department of Oral and Maxillofacial Surgery West China Hospital of Stomatology Sichuan University Chengdu China

**Keywords:** AMSTAR, GRADE, health‐related outcomes, sarcopenia, umbrella review

## Abstract

**Objective:**

The purpose of this umbrella review was to assess the associations between sarcopenia and adverse health‐related outcomes.

**Design:**

An umbrella review of meta‐analyses of observational studies.

**Setting and Participants:**

Patients with sarcopenia and controls without sarcopenia were included.

**Measures:**

The PubMed, Web of Science and Embase were searched for relevant systematic review and meta‐analysis. AMSTAR and GRADE system were used for methodological quality and evidence quality assessments, respectively.

**Results:**

Totally 54 outcomes extracted from 30 meta‐analyses were analyzed. Twenty out of 21 prognostic outcomes indicated that sarcopenia was significantly associated with poorer prognosis of gastric cancer, hepatocellular cancer, urothelial cancer, head and neck cancer, hematological malignancy, pancreatic cancer, breast cancer, colorectal cancer, lung cancer, esophageal cancer, and ovarian cancer. Besides, 10 out of 16 postoperative outcomes suggested that sarcopenia significantly increased the risk of multiple postoperative complications and prolonged the length of hospitalization of patients with digestive cancer. In age‐related outcomes, sarcopenia significantly increased the risk of dysphagia, cognitive impairment, fractures, falls, hospitalization, and all‐cause mortality of elderly populations. Moreover, sarcopenia was also associated with higher level of albuminuria, risk of depression, and several metabolic diseases.

**Conclusions and Implications:**

Sarcopenia significantly affected a wide range of adverse health‐related outcomes, particularly in patients of tumor and elderly populations. Because evidences of most outcomes were rated as “low” and “very low,” more prospective cohort studies are required in the future.

## Graphical abstract

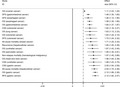

## 1 INTRODUCTION

Sarcopenia was first described as an age‐related decline in lean body mass in the 1980s.^1^ With sarcopenia research continuing for more than 30 years, recently the European Working Group on Sarcopenia in Older People (EWGSOP) revised the definition of sarcopenia as a progressive and generalized skeletal muscle disorder that is characterized by low muscle strength, low muscle quantity or quality, and low physical performance.^2^ Sarcopenia is a common disease worldwide, which is mainly associated with aging and older people, and it is also secondary to a systemic disease such as malignancy. It was suggested that the prevalence of sarcopenia was 10% in general elderly population worldwide.^3^ For specific populations, the prevalence of sarcopenia was 14.7% in hospitalized older patients, 41% to 59% in older nursing home residents, 12.9% to 40.4% in community living older adults, and 38.6% in cancer patients.^4^, ^5^, ^6^, ^7^

Sarcopenia is such a highly prevalent disease that might promote several adverse health‐related outcomes. Previous studies suggested that cancer patients with pre‐therapeutic sarcopenia had higher risk of postoperative complications, chemotherapy‐induced toxicity, and poorer survival than those without sarcopenia,^6^ and elderly people with sarcopenia were associated with functional decline, higher rate of hospitalizations, falls, and fractures.^8^ A few meta‐analyses have investigated the associations between sarcopenia and various health‐related outcomes, in which some results were inconsistent. For example, a meta‐analysis of seven studies^9^ suggested that sarcopenia was not associated with higher risk of major postoperative complications in patients of liver cancer, while another meta‐analysis of 28 studies^10^ indicated that sarcopenia significantly increased the risk of major postoperative complications in patients with gastrointestinal (GI) cancer. Recently, we also noticed an umbrella review that investigated the associations between sarcopenia and health‐related outcomes in older people.^11^ However, this umbrella review contained only six meta‐analyses with 14 outcomes, and current meta‐analyses about sarcopenia and prognostic outcomes of tumor, metabolic outcomes, and risk of depression were not included.

To better understand this issue, we systematically searched all the relevant meta‐analyses and provided an overview about the associations between sarcopenia and adverse health‐related outcomes in this study, and unified evidence assessments were also performed for all the outcomes reported currently.

## 2 METHODS

### 2.1 Literature search and eligibility criteria

For reviewing the existing meta‐analyses about sarcopenia and health‐related outcomes, we conducted this umbrella review according to the standardized procedures described previously.^12^, ^13^ The PubMed, Web of Science, and Embase were searched from the inception of the databases to April 2020. The following terms were used for search: (sarcopenia* OR sarcopenic* OR muscle*) AND (systematic review* OR meta‐analysis*), and detailed search strategies were shown in the Figure S1. Besides, we also reviewed the references of related studies for identifying potential meta‐analyses that were possibly missed in the initial search. Two authors reviewed the identified studies independently, and the inclusion criteria were: (a) published meta‐analysis or systematic review and meta‐analysis in English language, (b) investigating the associations between sarcopenia and health‐related outcomes, and (c) the summary effect size with 95% confidence intervals (CI) were reported. Systematic reviews without meta‐analysis and animal studies were excluded. All differences were discussed and resolved by consensus.

### 2.2 Data extraction

The data in each meta‐analysis were extracted by two authors independently. Briefly, the data we extracted were as follows: health‐related outcomes, the first author, year of publication, population characteristics, follow‐up, assessment of skeletal muscle, the number of studies and participants, metric of effect size, effects model of meta‐analysis, effect size with 95% CI, value of *I* ^2^, and publication bias. When a meta‐analysis contained multiple outcomes, each outcome would be extracted separately. Besides, if multiple meta‐analyses investigated a same outcome, usually we chose the newest meta‐analysis with the largest number of studies.

### 2.3 Methodological quality and evidence quality assessment

AMSTAR and the GRADE system were used for assessing the methodological quality of meta‐analysis and evidence quality of health‐related outcomes, respectively. AMSTAR was a measurement tool consisting of 11 items that has been shown to have good agreement, reliability, construct validity, and feasibility for methodological quality assessment,^14^, ^15^ and the GRADE system was an approach that offers a transparent and structured process for developing and presenting the summaries of evidence.^16^ In AMSTAR, the methodological quality was usually categorized as high (8‐11 items achieved), moderate (4‐7 items achieved), and low (0‐3 items achieved).^17^ In GRADE system, according to the assessment of risk of bias, inconsistence, indirectness, imprecision, and publication bias, the evidence quality was divided into four categories (high, moderate, low, and very low).^18^

### 2.4 Data analysis

Instead of searching the primary studies in meta‐analysis and reanalyzing the summary estimates with 95% CI, we just extracted the existing effect size and 95% CI for each health‐related outcome.^12^ When both random effects model and fixed effects model were performed for a same outcome, we primarily chose the one with random effects model as the final outcome. The value of *I* ^2^ and *P* value of Egger's or Begg's test in related meta‐analysis were extracted as the measures of heterogeneity and publication bias, respectively. If these data were lacked in meta‐analysis, we would calculate the *I^2^* statistic to assess heterogeneity when detailed original data were available, and we also performed the Egger's test for assessing the publication bias when the health‐related outcome contained at least 10 studies.^19^, ^20^ A value of *I^2^* > 50% was regarded as significant heterogeneity, and *P* value of <.1 for Egger's test indicated statistically significant publication bias. If *P* value of Egger's test <0.1, it could be an evidence of small‐study effects (whether smaller studies tend to give substantially larger estimates of effect size compared with larger studies) when the effect size of the largest study was more conservative than the summary effect size of the random effects meta‐analysis.^21^

## 3 RESULTS

### 3.1 Search results and study characteristics

We identified 3442 articles from PubMed, 10 480 articles from the Web of Science, and 3372 articles from Embase by the initial search. Additionally, nine articles were identified by reviewing the references of the related studies. Flowchart of the selection process was showed in Figure S2. Totally 54 studies met the inclusion criteria and were included for further assessment (references of the 54 studies were showed in supplementary material). Because there were several meta‐analyses investigating the same health‐related outcomes, we compared these meta‐analyses according to their publication year and number of included studies. Then, we chose the newest meta‐analysis with the largest number of studies. Finally, 54 health‐related outcomes extracted from 30 meta‐analyses^10^, ^22^, ^23^, ^24^, ^25^, ^26^, ^27^, ^28^, ^29^, ^30^, ^31^, ^32^, ^33^, ^34^, ^35^, ^36^, ^37^, ^38^, ^39^, ^40^, ^41^, ^42^, ^43^, ^44^, ^45^, ^46^, ^47^, ^48^, ^49^, ^50^ were reported in this umbrella review. These 54 outcomes were mainly about prognostic outcomes of tumor, postoperative outcomes, age‐related outcomes, metabolic outcomes, and other outcomes. Among the 54 outcomes, median number of included studies was 6 (range 2‐28), and the median number of participants was 1851 (range 485‐23 061) (Table 1).

**TABLE 1:** Associations between sarcopenia and adverse health‐related outcomes

Outcome	Author; year	Follow‐up	Assessment of skeletal muscle	No. of studies; participants	Metric of MA	Effects model	Effect size	95% CI	*I* ^2^ %	Publication bias	Small‐study effects	Quality of evidence
*Prognostic outcomes of tumor*												
OS (head and neck cancer)	Wong, A., et al, 2020	Range from 11 to 68 mo	CT	10; 2181	HR	REM	1.98	1.64‐2.39	0.00	Yes	Yes	Low
All‐cause mortality (breast cancer)	Zhang, X. M., et al, 2020	Range from 1.9 to 12 y	CT and DXA	6; 5497	HR	REM	1.71	1.25‐2.33	59.10	None	None	Low
Non‐relapse mortality (hematological malignancy)	Jia, S., et al, 2020	NR	CT	3; 1123	OR	REM	1.97	1.45‐2.68	0.00	NR	NR	Very low
OS (GI cancer)	Su, H., et al, 2019	NR	CT	20; 6232	HR	REM	1.60	1.37‐1.87	59.50	None	None	Low
DFS (GI cancer)	Su, H., et al, 2019	NR	CT	11; 4640	HR	FEM	1.46	1.30‐1.65	0.00	None	None	Moderate
OS (pancreatic cancer)	Bundred, J., et al, 2019	NR	CT and BIA	8; NR	OR	REM	1.95	1.35‐2.81	92.00	None	None	Very low
OS (gastric cancer)	Kamarajah, S. K., et al, 2019	NR	CT	9; 4236	HR	FEM	2.12	1.89‐2.38	37.00	None	None	Moderate
RFS (gastric cancer)	Kamarajah, S. K., et al, 2019	NR	CT	3; 1851	HR	FEM	2.12	1.82‐2.47	40.00	None	None	Low
CSS (gastric cancer)	Kamarajah, S. K., et al, 2019	NR	CT	3; 1741	HR	FEM	2.00	1.54‐2.59	0.00	None	None	Low
OS (esophageal cancer)	Deng, H. Y., et al, 2019	Range from 20 to 39.3 mo	CT	11; 1520	HR	FEM	1.58	1.35‐1.85	23.50	None	None	Moderate
DFS (esophageal cancer)	Deng, H. Y., et al, 2019	Range from 20 to 39.4 mo	CT	4; 561	HR	FEM	1.46	1.12‐1.90	0.00	NR	NR	Very low
OS (urothelial cancer)	Hu, X., et al, 2019	Range from 6 to 227 mo	CT	11; 1816	HR	REM	1.87	1.43‐2.45	54.30	None	None	Low
CSS (urothelial cancer)	Hu, X., et al, 2019	Range from 6 to 227 mo	CT	10; 1513	HR	REM	1.98	1.43‐2.75	39.40	None	None	Moderate
OS (lung cancer)	Deng, H. Y., et al, 2019	Range from 0 to 145 mo	CT	6; 1213	RR	REM	1.63	1.13‐2.33	73.10	None	None	Low
DFS (lung cancer)	Deng, H. Y., et al, 2019	Range from 0 to 146 mo	CT	3; 577	RR	REM	1.14	0.59‐2.17	72.10	NR	NR	Very low
OS (ovarian cancer)	Ubachs, J., et al, 2019	NR	CT	6; 1198	HR	FEM	1.11	1.03‐1.20	38.00	NR	NR	Low
OS (colorectal cancer)	Sun, G., et al, 2018	NR	CT	6; 4279	HR	REM	1.63	1.24‐2.14	48.40	None	None	Moderate
DFS (colorectal cancer)	Sun, G., et al, 2018	NR	CT	5; 1809	HR	REM	1.70	1.24‐2.32	31.20	NR	NR	Low
CSS (colorectal cancer)	Sun, G., et al, 2018	NR	CT	3; 2792	HR	REM	1.62	1.16‐2.27	17.60	NR	NR	Very low
All‐cause mortality (hepatocellular cancer)	Chang, K. V., et al, 2018	NR	CT	11; 2794	HR	REM	2.04	1.75‐2.38	<0.001	None	None	Moderate
Recurrence (hepatocellular cancer)	Chang, K. V., et al, 2018	NR	CT	6; 862	HR	REM	1.85	1.45‐2.38	<0.001	None	None	Low
*Postoperative outcomes*												
Postoperative pulmonary complications (esophageal cancer)	Wang, P. Y., et al, 2020	NR	CT and BIA	13; 2267	OR	REM	2.14	1.50‐3.04	46.40	None	None	Moderate
Anastomotic leakage (esophageal cancer)	Wang, P. Y., et al, 2020	NR	CT and BIA	12; 2163	OR	FEM	1.29	0.99‐1.67	7.90	None	None	Moderate
Overall postoperative complications (esophageal cancer)	Wang, P. Y., et al, 2020	NR	CT	11; 1972	OR	REM	1.42	1.08‐1.88	41.60	None	None	Moderate
Rate of readmission (digestive cancer)	Hua, H., et al, 2019	NR	CT and BIA	5; 919	RR	FEM	2.53	1.66‐3.85	0.00	NR	NR	Very low
Length of hospitalization (digestive cancer)	Hua, H., et al, 2019	NR	CT and BIA	9; 2174	RR	REM	4.61	1.84‐7.39	65.00	NR	NR	Very low
Major complications (GI cancer)	Simonsen, C., et al, 2018	NR	CT	28; 6883	RR	REM	1.40	1.20‐1.64	52.00	Yes	None	Very low
Major complications (patients of GI cancer with ERAS care)	Simonsen, C., et al, 2018	NR	CT	4; 703	RR	REM	1.29	0.91‐1.83	12.00	NR	NR	Very low
Major complications (patients of GI cancer without ERAS care)	Simonsen, C., et al, 2018	NR	CT	24; 6180	RR	REM	1.44	1.21‐1.71	56.00	Yes	None	Very low
Total complications (GI cancer)	Simonsen, C., et al, 2018	NR	CT	12; 3051	RR	REM	1.35	1.12‐1.61	60.00	None	None	Low
Postoperative pneumonia (gastric cancer)	Yang, Z., et al, 2018	NR	CT	6; 1563	OR	FEM	6.24	3.38‐11.51	0.00	NR	NR	Low
Postoperative ileus (gastric cancer)	Yang, Z., et al, 2018	NR	CT	5; 1464	OR	FEM	5.83	2.59‐13.08	21.00	NR	NR	Low
Postoperative intra‐abdominal infection (gastric cancer)	Yang, Z., et al, 2018	NR	CT	7; 1720	OR	FEM	1.15	0.64‐2.05	0.00	NR	NR	Low
Postoperative anastomotic leakage (gastric cancer)	Yang, Z., et al, 2018	NR	CT	7; 1720	OR	FEM	1.16	0.58‐2.33	0.00	NR	NR	Low
Postoperative delayed gastric emptying (gastric cancer)	Yang, Z., et al, 2018	NR	CT	4; 994	OR	FEM	1.22	0.45‐3.26	44.00	NR	NR	Very low
Postoperative infection (colorectal cancer)	Sun, G., et al, 2018	NA	CT	5; 1179	OR	REM	2.21	1.50‐3.25	0.00	NR	NR	Low
Postoperative anastomotic leakage (colorectal cancer)	Sun, G., et al, 2018	NA	CT	6; 2106	OR	REM	0.73	0.51‐1.05	0.00	NR	NR	Low
*Age‐related outcomes*												
Rate of hospitalization (people over 65 y old)	Zhao, Y., et al, 2019	Range from 0.5 to 7 y	BIA and DXA	8; 8174	RR	REM	1.40	1.04‐1.89	67.40	NR	NR	Very low
Rate of readmission (hospitalized people over 65 y old)	Zhao, Y., et al, 2019	Range from 0.5 to 3 y	BIA and DXA	4; 1302	RR	REM	1.75	1.01‐3.03	76.00	NR	NR	Very low
Length of hospitalization (community living people over 65 y old)	Zhao, Y., et al, 2019	Range from 3 to 7 y	BIA and DXA	4; 6276	OR	REM	1.21	0.90‐1.63	75.40	NR	NR	Very low
Risk of falls (community living people over 65 y old)	Yeung, S. S. Y., et al, 2019	NR	BIA and DXA	16; 23 061	OR	REM	1.75	1.55‐1.97	7.00	None	None	Moderate
Risk of falls (people over 60 y old in nursing home)	Zhang, X., et al, 2019	NR	BIA and DXA	3; 996	OR	FEM	1.12	0.84‐1.51	16.90	NR	NR	Very low
Risk of fractures (people over 65 y old)	Yeung, S. S. Y., et al, 2019	NR	BIA and DXA	12; 18 944	OR	REM	1.84	1.30‐2.62	91.00	None	None	Low
All‐cause mortality (elderly people in nursing home)	Zhang, X., et al, 2018	Range from 6 to 24 mo	BIA	6; 1494	HR	FEM	1.86	1.42‐2.45	0.00	None	None	Moderate
All‐cause mortality (community living people over 65 y old)	Liu, P., et al, 2017	Range from 3 to 14.4 mo	BIA and DXA	6; 7367	HR	REM	1.60	1.24‐2.06	27.80	None	None	Moderate
Risk of cognitive impairment (community living people over 60 y old)	Cabett Cipolli, G., et al, 2019	NR	NR	6; 7045	OR	REM	2.50	1.26‐4.92	84.00	NR	NR	Very low
Risk of dysphagia (people over 60 y old)	Zhao, W. T., et al, 2018	NR	CT and BIA	5; 913	OR	FEM	6.17	3.81‐10.00	15.97	NR	NR	Very low
*Metabolic outcomes*												
Hepatic encephalopathy (patients with liver cirrhosis)	Chang, K. V., et al, 2019	NR	CT	6; 1795	OR	REM	2.74	1.87‐4.01	54.97	Yes	Yes	Very low
Metabolic syndrome (middle‐aged and older nonobese adults)	Zhang, H., et al, 2018	NR	DXA	13; 4427	OR	REM	2.01	1.63‐2.47	79.20	None	None	Low
Steatohepatitis (patients with nonalcoholic fatty liver disease)	Yu, R., et al, 2018	NR	BIA and DXA	2; 534	OR	FEM	2.35	1.45‐3.81	0.00	NR	NR	Very low
Risk of nonalcoholic fatty liver disease	Pan, X., et al, 2018	NR	BIA and DXA	7; 18 654	OR	REM	1.29	1.12‐1.49	61.00	None	None	Low
Mortality of liver cirrhosis (patients with liver cirrhosis)	Kim, G., et al, 2017	NR	CT	4; 485	OR	REM	3.23	2.08‐5.01	32.00	None	None	Low
*Other outcomes*												
Albuminuria (patients with diabetes)	Ida, S., et al, 2019	NR	DXA	5; 1958	OR	REM	2.11	1.55‐2.88	45.00	NR	NR	Low
Risk of depression	Chang, K. V., et al, 2017	NR	BIA and DXA	10; 23 051	OR	REM	1.64	1.25‐2.16	64.38	Yes	Yes	Very low

Abbreviations: BIA, bioimpedance analysis; CI, confidence intervals; CSS, cancer‐specific survival; CT, computed tomography; DFS, disease‐free survival; DXA, dual x‐ray absorptiometry; FEM, fixed effects model; GI cancer, gastrointestinal cancer; HR, hazard ratios; MA, meta‐analysis; NR, not reported; OR, odds ratios; OS, overall survival; REM, random effects model; RFS, recurrence‐free survival; RR, relative risk.

### 3.2 Prognostic outcomes of tumor

There were totally 21 prognostic outcomes of over 12 kinds of tumors reported in this umbrella review^22^, ^23^, ^24^, ^25^, ^26^, ^27^, ^28^, ^29^, ^30^, ^31^, ^32^, ^36^ (Table 1). Associations between sarcopenia and overall survival (OS) were investigated in head and neck cancer, GI cancer, pancreatic cancer, gastric cancer, esophageal cancer, urothelial cancer, lung cancer, ovary cancer, and colorectal cancer, and sarcopenia was significantly associated with poorer OS of all these tumors. Besides, compared to those without sarcopenia, breast cancer and hepatocellular cancer patients with sarcopenia had 71% and 104% increased all‐cause mortality, respectively, and sarcopenia also increased the risk of recurrence of hepatocellular cancer (HR, 1.85; 95% CI 1.45‐2.38). Prognostic outcomes of disease‐free survival (DFS) were reported in four kinds of tumors, in which sarcopenia significantly decreased the DFS of GI cancer, esophageal cancer, and colorectal cancer, while no significant association was showed in lung cancer. Cancer‐specific survival (CSS) of gastric cancer, urothelial cancer, and colorectal cancer and recurrence‐free survival (RFS) of gastric cancer all had significantly inverse correlations with sarcopenia. For hematological malignancy, sarcopenia leaded to a 97% increment of non‐relapse mortality (OR, 1.97; 95% CI 1.45‐2.68).

**FIGURE 1:**
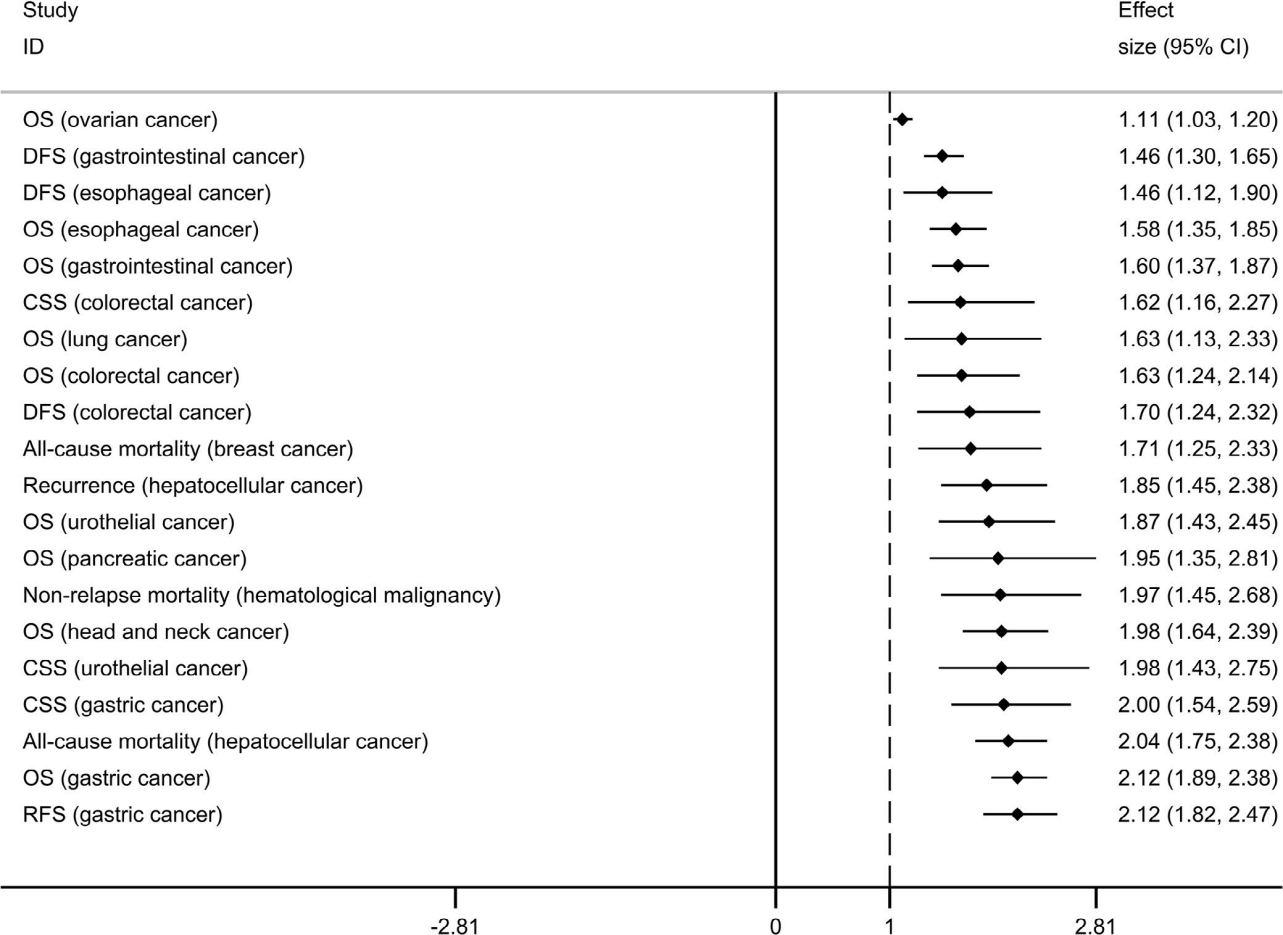
Forest plot of prognostic outcomes of tumor having significant associations with sarcopenia

In summary, among the 21 prognostic outcomes of tumor, 20 (95%) outcomes had significant associations with sarcopenia. According to the effect size, prognosis of gastric cancer was most affected by sarcopenia (Figure 1).

### 3.3 Postoperative outcomes

Totally 16 postoperative outcomes of tumors were reported.^10^, ^25^, ^33^, ^34^, ^35^ For esophageal cancer, patients with sarcopenia had significantly higher risk of overall postoperative complications and pulmonary complications, while no association was found with anastomotic leakage. In patients of digestive cancer, sarcopenia significantly increased the rate of readmission (RR, 2.53; 95% CI 1.66‐3.85) and prolonged the length of hospitalization (RR, 4.61; 95% CI 1.84‐7.39). Both major postoperative complications and total postoperative complications were increased by 40% and 35% in patients of GI cancer with sarcopenia, respectively. Moreover, subgroup analysis found that in patients of GI cancer with Enhanced Recovery after Surgery (ERAS) care, sarcopenia had no associations with the major complications (RR, 1.29; 95% CI 0.91‐1.83), whereas sarcopenia was also associated with increased major complications in those without ERAS care (RR, 1.44; 95% CI 1.21‐1.71). Additionally, sarcopenia was associated with increased postoperative pneumonia and ileus in patients of gastric cancer and increased postoperative infection in patients of colorectal cancer, respectively. However, no significant associations were showed between sarcopenia and postoperative intra‐abdominal infection, anastomotic leakage, and delayed gastric emptying in gastric cancer, and sarcopenia neither had association with postoperative anastomotic leakage in colorectal cancer.

**FIGURE 2:**
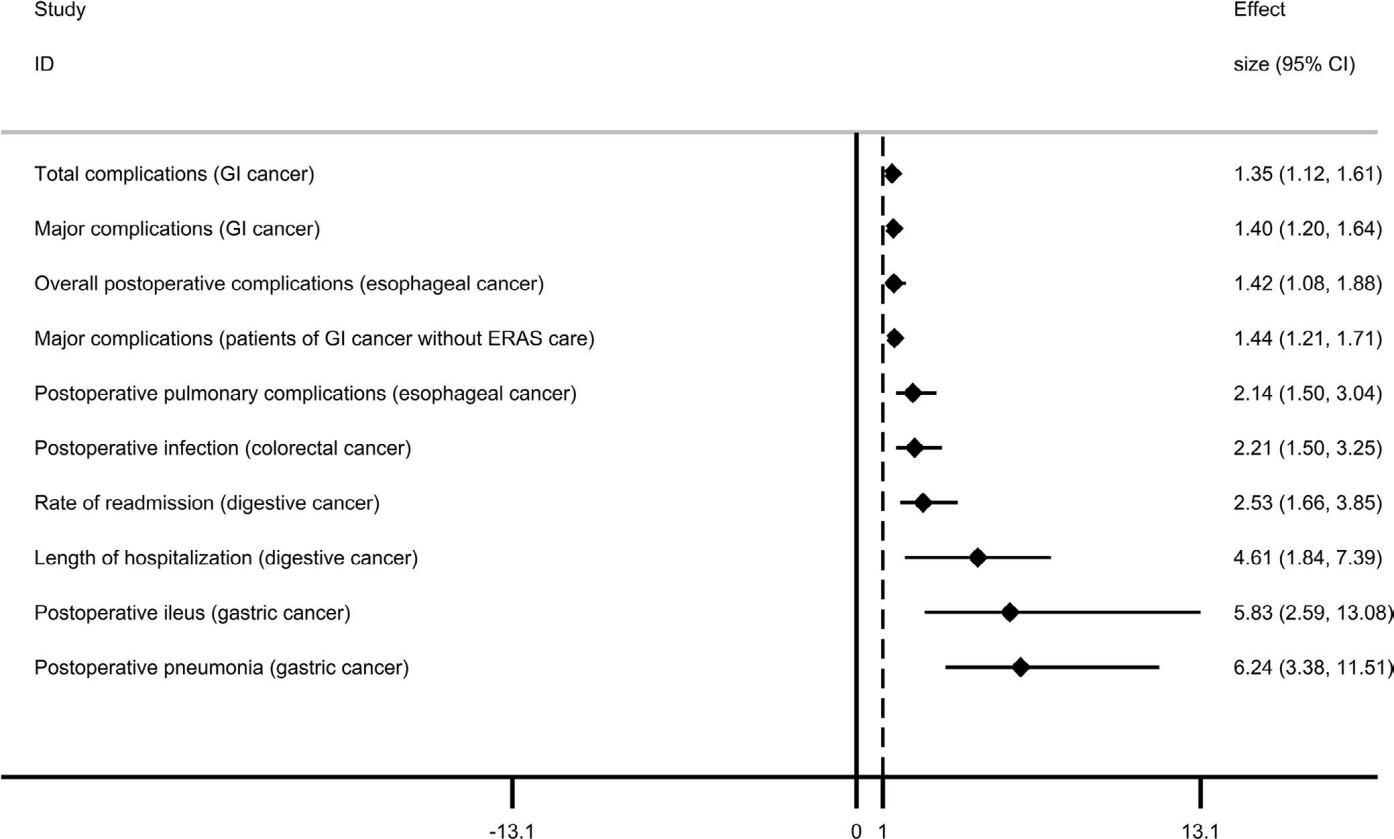
Forest plot of postoperative outcomes having significant associations with sarcopenia

In summary, 10 out of 16 postoperative outcomes (63%) had significant associations with sarcopenia. According to the effect size, total complications and major complications of GI cancer were comparatively less affected by sarcopenia, while the postoperative pneumonia and ileus of gastric cancer were most affected by sarcopenia (Figure 2).

### 3.4 Age‐related outcomes

There were totally 10 age‐related outcomes.^37^, ^38^, ^39^, ^40^, ^41^, ^42^, ^43^ In people over 65 years old, sarcopenia leaded to increased rate of hospitalization (RR 1.40, 95% CI 1.04‐1.89) and risk of fractures (OR 1.84, 95% CI 1.30‐2.62). Moreover, hospitalized people over 65 years old with sarcopenia had higher rate of readmission (RR 1.75, 95% CI 1.01‐3.03). In community living people over 65 years old, sarcopenia was associated with higher risk of falls and all‐cause mortality, while no association was showed with length of hospitalization. In people over 60 years old and community living people over 60 years old, those with sarcopenia had significantly higher risk of dysphagia and cognitive impairment, respectively. In nursing home, elderly people with sarcopenia had significantly higher all‐cause mortality, while there was no association between sarcopenia and risk of falls.

**FIGURE 3:**
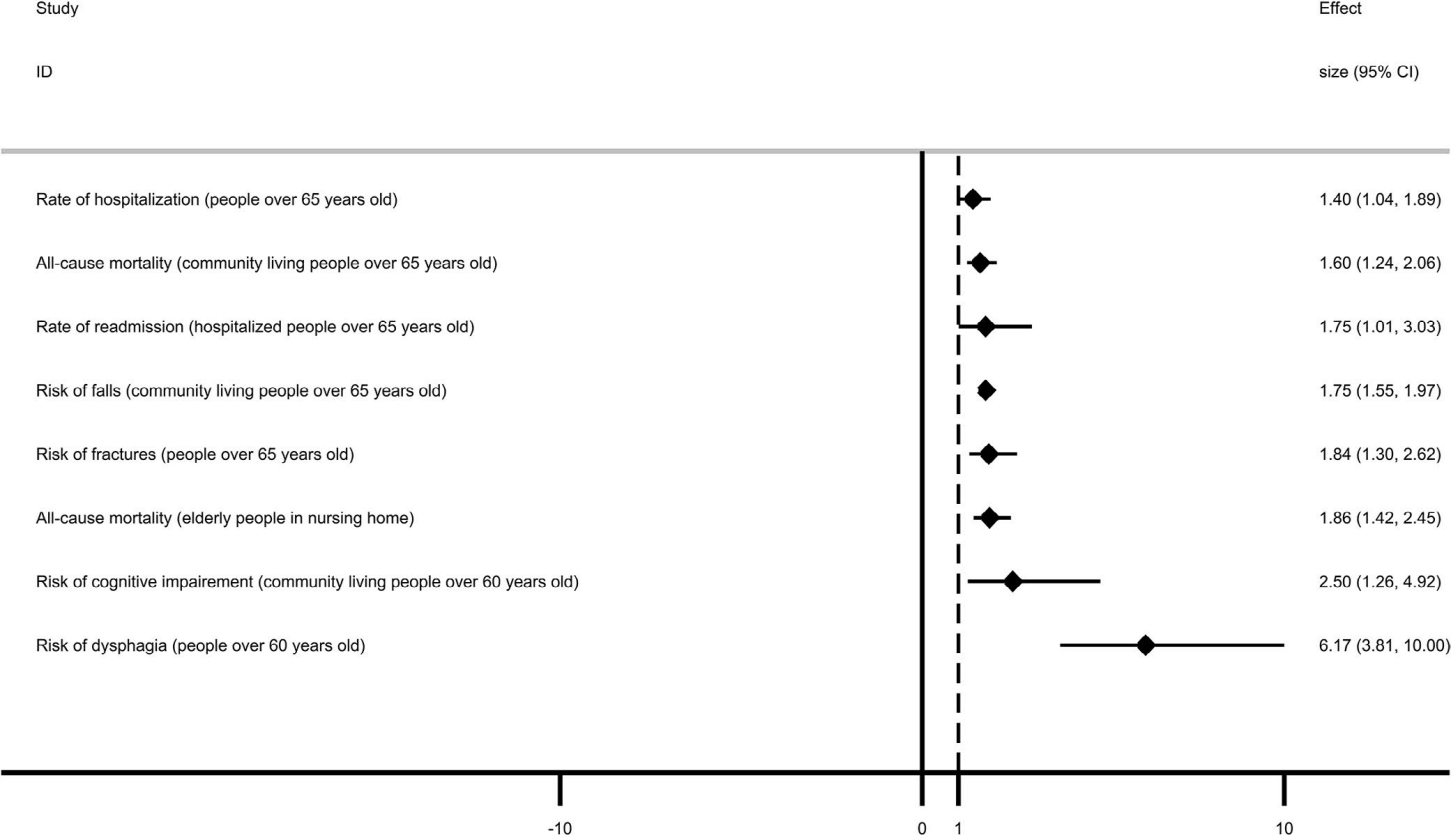
Forest plot of age‐related outcomes having significant associations with sarcopenia

In summary, eight out of 10 age‐related outcomes (80%) had significant associations with sarcopenia. Compared with people over 65 years old with sarcopenia in community, elderly people with sarcopenia in nursing home had higher all‐cause mortality. Moreover, the risk of dysphagia in people over 60 years old was most affected by sarcopenia (Figure 3).

### 3.5 Metabolic outcomes

**FIGURE 4:**
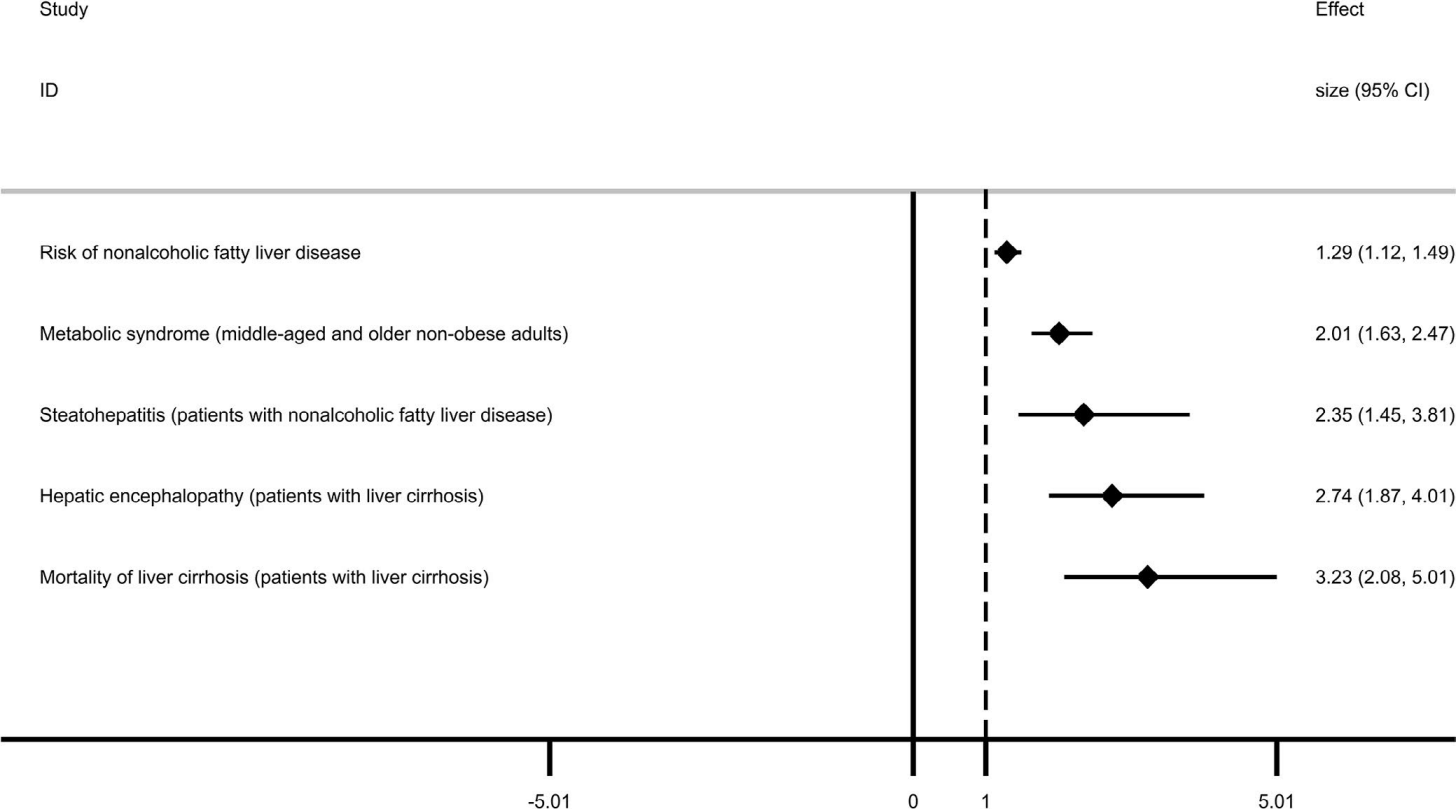
Forest plot of metabolic outcomes having significant associations with sarcopenia

Five meta‐analyses included in this study reported five metabolic outcomes.^44^, ^45^, ^46^, ^47^, ^48^ In middle‐aged and older nonobese adults, sarcopenia significantly increased the risk of metabolic syndrome (OR 2.01, 95% CI 1.63‐2.47). Besides, people with sarcopenia had a 29% increased risk of nonalcoholic fatty liver disease, and in patients with nonalcoholic fatty liver disease, sarcopenia was associated with higher risk of steatohepatitis (OR 2.35, 95% CI 1.45‐3.81). Sarcopenia also leaded to increased risk of hepatic encephalopathy and mortality in patients with liver cirrhosis. In summary, all the five metabolic outcomes had significant associations with sarcopenia, in which the mortality in patients with liver cirrhosis was most affected by sarcopenia (Figure 4).

### 3.6 Other outcomes

There were two single outcomes.^49^, ^50^ One reported that sarcopenia had positive correlation with albuminuria in patients with diabetes (OR 2.11, 95% CI 1.55‐2.88), and the other one showed that people with sarcopenia had higher risk of depression (OR 1.64, 95% CI 1.25‐2.16).

### 3.7 AMSTAR assessment and GRADE classification

The methodological quality of included meta‐analyses was assessed by AMSTAR which contained 11 items for scoring. Among the 30 included meta‐analyses, the median AMSTAR score was 8 (range 6‐11). Twenty‐two meta‐analyses (73%) had high methodological quality, and eight meta‐analyses (27%) had moderate methodological quality (Table S1).

Evidence quality assessment of the 54 health‐related outcomes was based on the GRADE system. Twelve outcomes (22%) were rated as “moderate,” 22 outcomes (41%) were rated as “low,” and 20 outcomes (37%) were rated as “very low.” Because all meta‐analyses in this umbrella review contained only observational studies, the risk of bias could be serious, and there was no outcome meeting a high quality of evidence. Moreover, high heterogeneity, small number of included studies or participants and significant publication bias also decreased the evidence quality of outcomes in this umbrella review. Detailed evidence quality assessments of the 54 outcomes were showed in Table S2.

## 4 DISCUSSION

In this umbrella review, we analyzed 30 current meta‐analyses and developed an overview of the associations between sarcopenia and 54 adverse health‐related outcomes. Particularly, the associations between sarcopenia and prognosis of tumor accounted for the largest percentage (39%) of the 54 outcomes. Although the evidences of majority prognostic outcomes were rated as “low” and “very low,” 95% of them had significant associations with sarcopenia, which indicating that sarcopenia was associated with poorer prognosis of diverse tumors. In postoperative outcomes, the tumors were mainly located at digestive tract, and sarcopenia was significantly associated with increased major postoperative complications, total postoperative complications, and several specific postoperative complications. Besides, about one thirds of specific postoperative outcomes were not associated with sarcopenia. Interestingly, we noticed that in patients of GI cancer with ERAS care, sarcopenia had no associations with the major postoperative complications. However, in patients of GI cancer without ERAS care, sarcopenia significantly increased the major postoperative complications. Although evidences of these two outcomes were rated as “very low,” we supposed that ERAS care might be helpful to improving the sarcopenia‐related postoperative complications, which needs more studies to verify in the future. Associations between sarcopenia and age‐related outcomes were also noticeable. Sarcopenia significantly affected a wide range of adverse outcomes such as all‐cause mortality, risk of falls, cognitive impairment, and dysphagia in different elderly populations, which seriously impaired the quality of life of the elderly. Moreover, sarcopenia was associated with several metabolic diseases and other outcomes including albuminuria and risk of depression in diverse populations, indicating that sarcopenia was a systematic medical condition and affected the human body more than the skeletal muscles themselves.

Sarcopenia was characterized by low muscle strength plus low muscle mass, so it might increase risk of falls and fractures in elderly people. Besides, decline of muscle function could affect the swallowing and breath and thereby increased the risk of dysphagia and postoperative pneumonia. Sarcopenia in cancer patients was commonly accompanied with malnutrition and disabled immune function, and it was also associated with higher chemotherapy toxicity and less efficacy of immunotherapy,^51^, ^52^ therefore, leading to higher postoperative complications and worse survival. In elderly people, some studies found that sarcopenia were closely associated with several comorbidities such as peptic ulcer disease, chronic obstructive pulmonary disease, osteoporosis, Parkinson's disease, and diabetes mellitus,^53^, ^54^, ^55^, ^56^, ^57^ which may explain why sarcopenia was associated with a wide range of age‐related outcomes such as higher all‐cause mortality, risk of hospitalization, readmission, and cognitive impairment. Skeletal muscle is an important organ for insulin‐mediated glucose uptake. Loss of skeletal muscle mass could lead to metabolism changes including decrease of insulin sensitivity, upregulation of gluconeogenesis, enhanced lipolysis, and generation of free fatty acids. Then, liver may take up the elevated fatty liver acids and excess glucose, which increased the risk of metabolic diseases.^58^, ^59^, ^60^

Current preventions and treatments for sarcopenia mainly included nutrition support and physical exercise. For healthy older populations, studies found that fish oil‐derived omega‐3 PUFA intake, high protein intake, resistance exercise training, and vitamin D3 supplements can be helpful for improving muscle mass and functions as well as preventing sarcopenia.^61^, ^62^, ^63^, ^64^, ^65^ Nitrate‐rich diets and oral nutritional support combined with exercise were also associated with better muscle functions.^66^, ^67^ Moreover, beta‐Hydroxy‐beta‐methylbutyrate supplements, high‐intensity resistance training, and dairy protein intake could be useful therapies for improving sarcopenia, and fat and fish dietary pattern might be associated with lower risk of sarcopenia in patients with GI cancer.^68^, ^69^, ^70^, ^71^ Although drug therapies such as testosterone, myostatin antibodies, and activin receptor antibodies might have potential effects on sarcopenia treatment,^72^ and recently a randomized controlled study reported that treatment with bimagrumab over 16 weeks increased muscle mass and strength in older adults with sarcopenia.^73^ Evidences of drug therapy for sarcopenia were still limited, and more studies about this issue are required.

There were several strengths in our study. We developed an overview of associations between sarcopenia and adverse health‐related outcomes in different populations. Totally we analyzed 30 meta‐analyses and reported 54 outcomes. The methodological quality of included studies and evidence quality of reported outcomes were assessed by unified method, and we found that sarcopenia significantly affected a wide range of adverse health‐related outcomes. There were also some limitations in this study. Meta‐analyses in this umbrella review contained only observational studies, which could decrease the quality of evidence. Besides, the methods for assessing the skeletal muscle were inconsistent, and CT, BIA, and DXA were applied in different meta‐analyses, which might increase the risk of bias.

## 5 CONCLUSIONS AND IMPLICATIONS

In conclusion, sarcopenia significantly affected a wide range of adverse health‐related outcomes, particularly in patients of tumor and elderly populations. Besides, associations between sarcopenia and risk of metabolic diseases, depression and albuminuria were also noticeable. Considering that evidences of most outcomes were rated as “low” and “very low,” more prospective cohort studies are required in the future.

## CONFLICT OF INTEREST

There was no conflict of interest.

## AUTHOR CONTRIBUTIONS

LX, RZ, and QYW contributed equally in this study. LX, RZ, QYW, YZ, YW, YPC, and XDS contributed to the data collection and analysis. LX, RZ, QYW, and YTW wrote the manuscript under the guidance of XTW. All the authors have read manuscript, and XTW approved the final manuscript.

## ETHICAL APPROVAL

This is an umbrella review of meta‐analysis, and ethical approval is not applicable.

## Supporting information

Supplementary Material

## Data Availability

All data generated or analyzed during this study are included in this published article.
