# Supplementary material for: Sarcopenia and adverse health‐related outcomes: An umbrella review of meta‐analyses of observational studies
Source: Cancer Med. 2020 Sep 13;9(21):7964–78. doi: 10.1002/cam4.3428 (PMC7643685; doi:10.1002/cam4.3428)
Supplement: Supplementary file 1 — Supplementary Material [file CAM4-9-7964-s001.pdf]

Figure S1. Search strategies

PubMed:

History

Download history Clear history

Search	Add to builder	Query	Items found	Time
#3	Add	Search ((((Sarcopenia[Title/Abstract]) OR sarcopenic[Title/Abstract]) OR muscle[Title/Abstract])) AND ((Systematic review[Title/Abstract]) OR meta-analysis[Title/Abstract])	3442	08:33:59
#2	Add	Search (Systematic review[Title/Abstract]) OR meta-analysis[Title/Abstract]	213086	08:33:23
#1	Add	Search ((Sarcopenia[Title/Abstract]) OR sarcopenic[Title/Abstract]) OR muscle[Title/Abstract]	620947	08:32:37

Web of Science:

Set	Results	Save History / Open Saved History	Combine Sets (AND / OR, Combine)	Delete Sets (Select All, Delete)
# 8	10,480	#7 AND #6<br>*Databases= WOS, DIIDW, INSPEC, KJD, MEDLINE, RSCI, SCIELO Timespan=All years*<br>*Search language=Auto*		
# 7	405,754	#5 OR #4<br>*Databases= WOS, DIIDW, INSPEC, KJD, MEDLINE, RSCI, SCIELO Timespan=All years*<br>*Search language=Auto*		
# 6	1,912,418	#3 OR #2 OR #1<br>*Databases= WOS, DIIDW, INSPEC, KJD, MEDLINE, RSCI, SCIELO Timespan=All years*<br>*Search language=Auto*		
# 5	209,507	TOPIC: (meta-analysis)<br>*Databases= WOS, DIIDW, INSPEC, KJD, MEDLINE, RSCI, SCIELO Timespan=All years*<br>*Search language=Auto*		
# 4	276,758	TOPIC: (Systematic review)<br>*Databases= WOS, DIIDW, INSPEC, KJD, MEDLINE, RSCI, SCIELO Timespan=All years*<br>*Search language=Auto*		
# 3	1,909,482	TOPIC: (muscle)<br>*Databases= WOS, DIIDW, INSPEC, KJD, MEDLINE, RSCI, SCIELO Timespan=All years*<br>*Search language=Auto*		
# 2	2,239	TOPIC: (sarcopenic)<br>*Databases= WOS, DIIDW, INSPEC, KJD, MEDLINE, RSCI, SCIELO Timespan=All years*<br>*Search language=Auto*		
# 1	13,288	TOPIC: (Sarcopenia)<br>*Databases= WOS, DIIDW, INSPEC, KJD, MEDLINE, RSCI, SCIELO Timespan=All years*<br>*Search language=Auto*		
			AND OR Combine	Select All Delete

Embase:

▾ Search History (3) View Saved

# ▲	Searches	Results	Type	Actions	Annotations
1	(Sarcopenia or sarcopenic or muscle).ab.	702709	Advanced	Display Results More ▾	
2	(Systematic review or meta-analysis).ab.	211500	Advanced	Display Results More ▾	
3	1 and 2	3372	Advanced	Display Results More ▾	

Figure S2. Flowchart of the selection process

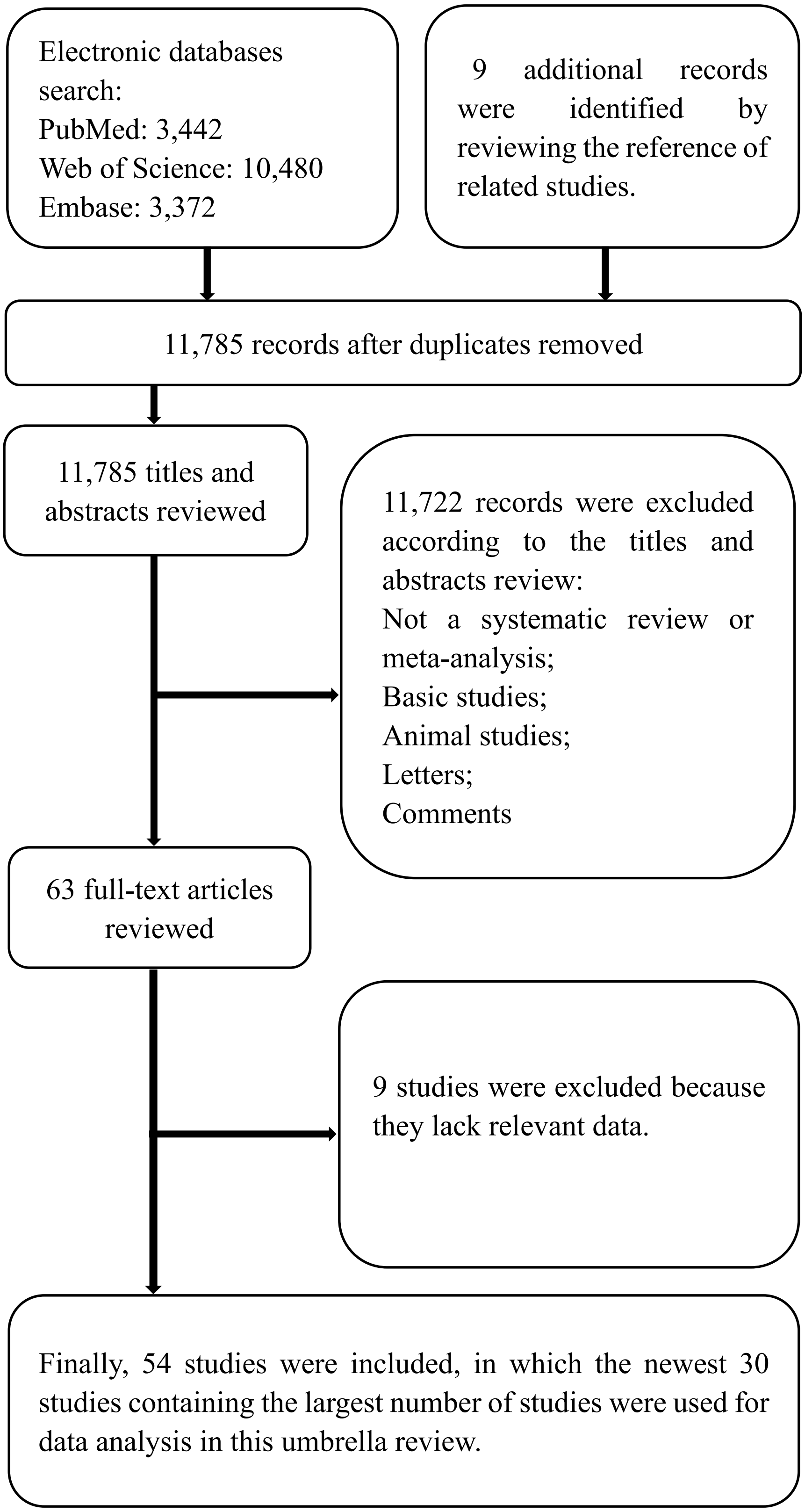

Table S1. Methodological quality assessment of included meta-analyses according to the AMSTAR

Author	Year	No. of 1	No. of 2	No. of 3	No. of 4	No. of 5	No. of 6	No. of 7	No. of 8	No. of 9	No. of 10	No. of 11	AMSTAR
Wong, A., et al	2020	1	1	1	1	1	1	1	1	1	1	1	11
Zhang, X. M., et al	2020	1	1	1	1	1	1	1	1	1	1	1	11
Jia, S., et al	2020	1	1	1	1	1	1	1	1	1	0	1	10
Wang, P. Y., et al	2020	1	0	1	0	1	1	1	1	1	1	1	9
Bundred, J., et al	2019	1	1	1	1	1	0	1	1	1	1	1	10
Ida, S., et al	2019	0	0	1	1	1	1	1	1	1	0	1	8
Su, H., et al	2019	0	0	1	1	1	1	1	1	1	0	1	8
Ubachs, J., et al	2019	0	1	1	0	1	1	1	1	1	0	1	8
Zhao, Y., et al	2019	0	0	0	1	0	1	1	1	1	0	1	6
Deng, H. Y., et al	2019	0	1	1	1	0	1	1	1	1	1	1	9
Hu, X., et al	2019	0	0	1	1	0	1	1	1	1	1	1	8
Deng, H. Y., et al	2019	0	1	1	1	1	1	1	1	1	0	1	9
Kamarajah, S. K., et al	2019	0	0	1	0	0	1	1	1	1	1	1	7
Hua, H., et al	2019	0	1	0	1	0	1	1	1	1	0	1	7
Chang, K. V., et al	2019	0	0	1	1	0	1	1	1	1	1	1	8
Yeung, S. S. Y., et al	2019	1	0	1	1	0	1	1	1	1	1	1	9
Zhang, X., et al	2019	1	1	1	1	0	1	1	1	1	0	1	9
Cabett Cipolli, G., et al	2019	0	1	1	1	0	1	1	1	1	0	1	8
Chang, K. V., et al	2018	0	1	1	1	0	1	1	1	1	1	1	9
Sun, G., et al	2018	0	1	0	1	0	1	1	1	1	1	1	8
Yang, Z., et al	2018	0	1	0	0	1	1	1	1	1	0	1	7

Simonsen, C., et al	2018	0	1	1	0	1	1	1	1	1	1	1	9
Zhang, H., et al	2018	0	1	0	1	0	1	0	1	1	1	1	7
Yu, R., et al	2018	0	1	1	0	0	1	1	1	1	0	1	7
Pan, X., et al	2018	0	1	1	1	0	1	1	1	1	1	1	9
Zhao, W. T., et al	2018	0	1	1	1	0	0	1	1	1	0	1	7
Zhang, X., et al	2018	0	1	1	1	0	1	1	1	1	1	1	9
Kim, G., et al	2017	0	1	1	1	0	1	1	1	1	1	1	9
Chang, K. V., et al	2017	0	0	1	0	0	1	1	1	1	1	1	7
Liu, P., et al	2017	0	0	1	1	0	1	1	1	1	1	1	8

Table S2. Evidence quality assessment of included health-related outcomes according to the GRADE

Outcome	Author; year	Risk of bias	Inconsistency	Indirectness	Imprecision	Publication bias	Quality of evidence
OS (head and neck cancer)	Wong, A., et al; 2020	Serious	Not serious	Not serious	Not serious	Serious	Low
All-cause mortality （breast cancer）	Zhang, X. M., et al; 2020	Serious	Serious	Not serious	Not serious	Not serious	Low
Non-relapse mortality (hematological malignancy)	Jia, S., et al; 2020	Serious	Not serious	Not serious	Serious	Serious	Very low
OS (gastrointestinal cancer)	Su, H., et al; 2019	Serious	Serious	Not serious	Not serious	Not serious	Low
DFS (gastrointestinal cancer)	Su, H., et al; 2019	Serious	Not serious	Not serious	Not serious	Not serious	Moderate
OS (pancreatic cancer)	Bundred, J., et al; 2019	Serious	Serious	Not serious	Serious	Not serious	Very low
OS (gastric cancer)	Kamarajah, S. K., et al; 2019	Serious	Not serious	Not serious	Not serious	Not serious	Moderate
RFS (gastric cancer)	Kamarajah, S. K., et al; 2019	Serious	Not serious	Not serious	Serious	Not serious	Low
CSS (gastric cancer)	Kamarajah, S. K., et al; 2019	Serious	Not serious	Not serious	Serious	Not serious	Low
OS (esophageal cancer)	Deng, H. Y., et al; 2019	Serious	Not serious	Not serious	Not serious	Not serious	Moderate
DFS (esophageal cancer)	Deng, H. Y., et al; 2019	Serious	Not serious	Not serious	Serious	Serious	Very low
OS (urothelial carcinoma)	Hu, X., et al; 2019	Serious	Serious	Not serious	Not serious	Not serious	Low

CSS (urothelial carcinoma)	Hu, X., et al; 2019	Serious	Not serious	Not serious	Not serious	Not serious	Moderate
OS (lung cancer)	Deng, H. Y., et al; 2019	Serious	Serious	Not serious	Not serious	Not serious	Low
DFS (lung cancer)	Deng, H. Y., et al; 2019	Serious	Serious	Not serious	Serious	Serious	Very low
OS (ovarian cancer)	Ubachs, J., et al; 2019	Serious	Not serious	Not serious	Not serious	Serious	Low
OS (colorectal cancer)	Sun, G., et al; 2018	Serious	Not serious	Not serious	Not serious	Not serious	Moderate
DFS (colorectal cancer)	Sun, G., et al; 2018	Serious	Not serious	Not serious	Not serious	Serious	Low
CSS (colorectal cancer)	Sun, G., et al; 2018	Serious	Not serious	Not serious	Serious	Serious	Very low
All-cause mortality (hepatocellular carcinoma)	Chang, K. V., et al; 2018	Serious	Not serious	Not serious	Not serious	Not serious	Moderate
Recurrence (hepatocellular carcinoma)	Chang, K. V., et al; 2018	Serious	Not serious	Not serious	Serious	Not serious	Low
Postoperative pulmonary complications (esophageal cancer)	Wang, P. Y., et al; 2020	Serious	Not serious	Not serious	Not serious	Not serious	Moderate
Anastomotic leakage (esophageal cancer)	Wang, P. Y., et al; 2020	Serious	Not serious	Not serious	Serious	Not serious	Low
Overall postoperative complications (esophageal cancer)	Wang, P. Y., et al; 2020	Serious	Not serious	Not serious	Not serious	Not serious	Moderate
Rate of readmission (digestive carcinoma)	Hua, H., et al; 2019	Serious	Not serious	Not serious	Serious	Serious	Very low
Length of hospitalization (digestive carcinoma)	Hua, H., et al; 2019	Serious	Serious	Not serious	Not serious	Serious	Very low

Major complications (GI cancer)	Simonsen, C., et al; 2018	Serious	Serious	Not serious	Not serious	Serious	Very low
Major complications (patients of GI cancer with ERAS care)	Simonsen, C., et al; 2018	Serious	Not serious	Not serious	Serious	Serious	Very low
Major complications (patients of GI cancer without ERAS care)	Simonsen, C., et al; 2018	Serious	Serious	Not serious	Not serious	Serious	Very low
Total complications (GI cancer)	Simonsen, C., et al; 2018	Serious	Serious	Not serious	Not serious	Not serious	Low
Postoperative pneumonia (gastric cancer)	Yang, Z., et al; 2018	Serious	Not serious	Not serious	Not serious	Serious	Low
Postoperative ileus (gastric cancer)	Yang, Z., et al; 2018	Serious	Not serious	Not serious	Not serious	Serious	Low
Postoperative intra-abdominal infection (gastric cancer)	Yang, Z., et al; 2018	Serious	Not serious	Not serious	Serious	Serious	Very low
Postoperative anastomotic leakage (gastric cancer)	Yang, Z., et al; 2018	Serious	Not serious	Not serious	Serious	Serious	Very low
Postoperative delayed gastric emptying (gastric cancer)	Yang, Z., et al; 2018	Serious	Not serious	Not serious	Serious	Serious	Very low
Postoperative infection (colorectal cancer)	Sun, G., et al; 2018	Serious	Not serious	Not serious	Not serious	Serious	Low
Postoperative anastomotic leakage (colorectal cancer)	Sun, G., et al; 2018	Serious	Not serious	Not serious	Serious	Serious	Very low
Rate of hospitalization (people over 65 years old)	Zhao, Y., et al; 2019	Serious	Serious	Not serious	Not serious	Serious	Very low
Rate of readmission (hospitalized people over 65 years old)	Zhao, Y., et al; 2019	Serious	Serious	Not serious	Serious	Serious	Very low
Length of hospitalization (community living people over 65 years old)	Zhao, Y., et al; 2019	Serious	Serious	Not serious	Serious	Serious	Very low

Risk of falls (community living people over 65 years old)	Yeung, S. S. Y., et al; 2019	Serious	Not serious	Not serious	Not serious	Not serious	Moderate
Risk of falls (people over 60 years old in nursing home)	Zhang, X., et al; 2019	Serious	Not serious	Not serious	Serious	Serious	Very low
Risk of fractures (people over 65 years old)	Yeung, S. S. Y., et al; 2019	Serious	Serious	Not serious	Not serious	Not serious	Low
All-cause mortality (elderly people in nursing home)	Zhang, X., et al; 2018	Serious	Not serious	Not serious	Not serious	Not serious	Moderate
All-cause mortality (community living people over 65 years old)	Liu, P., et al; 2017	Serious	Not serious	Not serious	Not serious	Not serious	Moderate
Risk of cognitive impairment (community living people over 60 years old)	Cabett Cipolli, G., et al; 2019	Serious	Serious	Not serious	Not serious	Serious	Very low
Risk of dysphagia (people over 60 years old)	Zhao, W. T., et al; 2018	Serious	Not serious	Not serious	Serious	Serious	Very low
Hepatic encephalopathy (patients with liver cirrhosis)	Chang, K. V., et al; 2019	Serious	Serious	Not serious	Not serious	Serious	Very low
Metabolic syndrome (middle-aged and older non-obese adults)	Zhang, H., et al; 2018	Serious	Serious	Not serious	Not serious	Not serious	Low
Steatohepatitis (patients with nonalcoholic fatty liver disease)	Yu, R., et al; 2018	Serious	Not serious	Not serious	Serious	Serious	Very low
Risk of nonalcoholic fatty liver disease	Pan, X., et al; 2018	Serious	Serious	Not serious	Not serious	Not serious	Low
Mortality of liver cirrhosis (patients with liver cirrhosis)	Kim, G., et al; 2017	Serious	Not serious	Not serious	Serious	Not serious	Low
Albuminuria (patients with diabetes)	Ida, S., et al; 2019	Serious	Not serious	Not serious	Not serious	Serious	Low
Risk of depression	Chang, K. V., et al; 2017	Serious	Serious	Not serious	Not serious	Serious	Very low

References of the 54 studies meeting the inclusion criteria of this umbrella review:

1. Zhang XM, Dou QL, Zeng Y, Yang Y, Cheng ASK, Zhang WW. Sarcopenia as a predictor of mortality in women with breast cancer: a meta-analysis and systematic review. BMC Cancer. Mar 4 2020;20(1):172.

2. Wong A, Zhu D, Kraus D, Tham T. Radiologically Defined Sarcopenia Affects Survival in Head and Neck Cancer: A Meta-Analysis. Laryngoscope. Mar 27 2020.

3. Wang PY, Xu LD, Chen XK, et al. Sarcopenia and Short-Term Outcomes After Esophagectomy: A Meta-analysis. Ann Surg Oncol. Feb 10 2020.

4. Schizas D, Frountzas M, Lidoriki I, et al. Sarcopenia does not affect postoperative complication rates in oesophageal cancer surgery: a systematic review and meta-analysis. Ann R Coll Surg Engl. Feb 2020;102(2):120-132.

5. Papaconstantinou D, Vretakakou K, Paspala A, et al. The impact of preoperative sarcopenia on postoperative complications following esophagectomy for esophageal neoplasia: a systematic review and meta-analysis. Dis Esophagus. Mar 20 2020.

6. McSharry V, Mullee A, McCann L, Rogers AC, McKiernan M, Brennan DJ. The Impact of Sarcopenia and Low Muscle Attenuation on Overall Survival in Epithelial Ovarian Cancer: A Systematic Review and Meta-analysis. Ann Surg Oncol. Mar 27 2020.

7. Jia S, Qiao R, Xiao Y, et al. Prognostic value of sarcopenia in survivors of hematological malignances undergoing a hematopoietic stem cell transplantation: a systematic review and meta-analysis. Support Care Cancer. Feb 23 2020.

8. Guo Z, Gu C, Gan S, et al. Sarcopenia as a predictor of postoperative outcomes after urologic oncology surgery: A systematic review and meta-analysis. Urol Oncol. Apr 5 2020.

9. Chen H, Ma J, Liu A, Cui Y, Ma X. The association between sarcopenia and fracture in middle-aged and elderly people: A systematic review and meta-analysis of cohort studies. Injury. Feb 18 2020.

10. Zhao Y, Zhang Y, Hao Q, Ge M, Dong B. Sarcopenia and hospital-related outcomes in the old people: a systematic review and meta-analysis. Aging Clin Exp Res. Jan 2019;31(1):5-14.

11. Zhang X, Huang P, Dou Q, et al. Falls among older adults with sarcopenia dwelling in nursing home or community: A meta-analysis. Clin Nutr. Jan 8 2019.

12. Yeung SSY, Reijnierse EM, Pham VK, et al. Sarcopenia and its association with falls and fractures in older adults: A systematic review and meta-analysis. J Cachexia

Sarcopenia Muscle. Jun 2019;10(3):485-500.
13. Yang M, Shen Y, Tan L, Li W. Prognostic Value of Sarcopenia in Lung Cancer: A Systematic Review and Meta-analysis. Chest. May 22 2019.
14. Ubachs J, Ziemons J, Minis-Rutten IJG, et al. Sarcopenia and ovarian cancer survival: a systematic review and meta-analysis. J Cachexia Sarcopenia Muscle. Dec 2019;10(6):1165-1174.
15. Su H, Ruan J, Chen T, Lin E, Shi L. CT-assessed sarcopenia is a predictive factor for both long-term and short-term outcomes in gastrointestinal oncology patients: a systematic review and meta-analysis. Cancer imaging : the official publication of the International Cancer Imaging Society. Dec 3 2019;19(1):82.
16. Peng TC, Chen WL, Wu LW, Chang YW, Kao TW. Sarcopenia and cognitive impairment: A systematic review and meta-analysis. Clin Nutr. Dec 17 2019.
17. Li J, Deng Y, Zhang M, Cheng Y, Zhao X, Ji Z. Prognostic value of radiologically determined sarcopenia prior to treatment in urologic tumors: A meta-analysis. Medicine (Baltimore). Sep 2019;98(38):e17213.
18. Kamarajah SK, Bundred J, Tan BHL. Body composition assessment and sarcopenia in patients with gastric cancer: a systematic review and meta-analysis. Gastric Cancer. Jan 2019;22(1):10-22.
19. Ida S, Kaneko R, Imataka K, Murata K. Association between Sarcopenia and Renal Function in Patients with Diabetes: A Systematic Review and Meta-Analysis. J Diabetes Res. 2019;2019:1365189.
20. Hua H, Xu X, Tang Y, Ren Z, Xu Q, Chen L. Effect of sarcopenia on clinical outcomes following digestive carcinoma surgery: a meta-analysis. Support Care Cancer. Jul 2019;27(7):2385-2394.
21. Hu X, Dou WC, Shao YX, et al. The prognostic value of sarcopenia in patients with surgically treated urothelial carcinoma: A systematic review and meta-analysis. Eur J Surg Oncol. May 2019;45(5):747-754.
22. Eros A, Soos A, Hegyi P, et al. Sarcopenia as an independent predictor of the surgical outcomes of patients with inflammatory bowel disease: a meta-analysis. Surg Today. Oct 15 2019.
23. Deng HY, Zha P, Peng L, Hou L, Huang KL, Li XY. Preoperative sarcopenia is a predictor of poor prognosis of esophageal cancer after esophagectomy: a comprehensive systematic review and meta-analysis. Dis Esophagus. Mar 1 2019;32(3).
24. Deng HY, Hou L, Zha P, Huang KL, Peng L. Sarcopenia is an independent

unfavorable prognostic factor of non-small cell lung cancer after surgical resection: A comprehensive systematic review and meta-analysis. Eur J Surg Oncol. May 2019;45(5):728-735.
25. Chang KV, Chen JD, Wu WT, Huang KC, Lin HY, Han DS. Is sarcopenia associated with hepatic encephalopathy in liver cirrhosis? A systematic review and meta-analysis. J Formos Med Assoc. Apr 2019;118(4):833-842.
26. Cabett Cipolli G, Sanches Yassuda M, Aprahamian I. Sarcopenia Is Associated with Cognitive Impairment in Older Adults: A Systematic Review and Meta-Analysis. J Nutr Health Aging. 2019;23(6):525-531.
27. Bundred J, Kamarajah SK, Roberts KJ. Body composition assessment and sarcopenia in patients with pancreatic cancer: a systematic review and meta-analysis. HPB (Oxford). Dec 2019;21(12):1603-1612.
28. Antoniou GA, Rojoa D, Antoniou SA, Alfahad A, Torella F, Juszczak MT. Effect of Low Skeletal Muscle Mass on Post-operative Survival of Patients With Abdominal Aortic Aneurysm: A Prognostic Factor Review and Meta-Analysis of Time-to-Event Data. Eur J Vasc Endovasc Surg. Jun 13 2019.
29. Zhao WT, Yang M, Wu HM, Yang L, Zhang XM, Huang Y. Systematic Review and Meta-Analysis of the Association between Sarcopenia and Dysphagia. J Nutr Health Aging. 2018;22(8):1003-1009.
30. Zhang Y, Hao Q, Ge M, Dong B. Association of sarcopenia and fractures in community-dwelling older adults: a systematic review and meta-analysis of cohort studies. Osteoporos Int. Jun 2018;29(6):1253-1262.
31. Zhang X, Zhang W, Wang C, Tao W, Dou Q, Yang Y. Sarcopenia as a predictor of hospitalization among older people: a systematic review and meta-analysis. BMC Geriatr. Aug 22 2018;18(1):188.
32. Zhang X, Wang C, Dou Q, Zhang W, Yang Y, Xie X. Sarcopenia as a predictor of all-cause mortality among older nursing home residents: a systematic review and meta-analysis. Bmj Open. Nov 12 2018;8(11):e021252.
33. Zhang H, Lin S, Gao T, et al. Association between Sarcopenia and Metabolic Syndrome in Middle-Aged and Older Non-Obese Adults: A Systematic Review and Meta-Analysis. Nutrients. Mar 16 2018;10(3).
34. Yu R, Shi Q, Liu L, Chen L. Relationship of sarcopenia with steatohepatitis and advanced liver fibrosis in non-alcoholic fatty liver disease: a meta-analysis. Bmc Gastroenterol. Apr 19 2018;18(1):51.

35. Yang Z, Zhou X, Ma B, Xing Y, Jiang X, Wang Z. Predictive Value of Preoperative Sarcopenia in Patients with Gastric Cancer: a Meta-analysis and Systematic Review. J Gastrointest Surg. Nov 2018;22(11):1890-1902.
36. Wijarnpreecha K, Panjawatanan P, Thongprayoon C, Jaruvongvanich V, Ungprasert P. Sarcopenia and risk of nonalcoholic fatty liver disease: A meta-analysis. Saudi J Gastroenterol. Jan-Feb 2018;24(1):12-17.
37. Sun G, Li Y, Peng Y, et al. Can sarcopenia be a predictor of prognosis for patients with non-metastatic colorectal cancer? A systematic review and meta-analysis. Int J Colorectal Dis. Oct 2018;33(10):1419-1427.
38. Simonsen C, de Heer P, Bjerre ED, et al. Sarcopenia and Postoperative Complication Risk in Gastrointestinal Surgical Oncology: A Meta-analysis. Ann Surg. Jul 2018;268(1):58-69.
39. Ratnayake CB, Loveday BP, Shrikhande SV, Windsor JA, Pandanaboyana S. Impact of preoperative sarcopenia on postoperative outcomes following pancreatic resection: A systematic review and meta-analysis. Pancreatology. Dec 2018;18(8):996-1004.
40. Pan X, Han Y, Zou T, et al. Sarcopenia Contributes to the Progression of Nonalcoholic Fatty Liver Disease- Related Fibrosis: A Meta-Analysis. Dig Dis. 2018;36(6):427-436.
41. Mintziras I, Miligkos M, Wachter S, Manoharan J, Maurer E, Bartsch DK. Sarcopenia and sarcopenic obesity are significantly associated with poorer overall survival in patients with pancreatic cancer: Systematic review and meta-analysis. Int J Surg. Nov 2018;59:19-26.
42. Hajibandeh S, Hajibandeh S, Jarvis R, Bhogal T, Dalmia S. Meta-analysis of the effect of sarcopenia in predicting postoperative mortality in emergency and elective abdominal surgery. Surgeon. Oct 30 2018.
43. Du Y, Oh C, No J. Associations between Sarcopenia and Metabolic Risk Factors: A Systematic Review and Meta-Analysis. J Obes Metab Syndr. Sep 30 2018;27(3):175-185.
44. Chang KV, Chen JD, Wu WT, Huang KC, Hsu CT, Han DS. Association between Loss of Skeletal Muscle Mass and Mortality and Tumor Recurrence in Hepatocellular Carcinoma: A Systematic Review and Meta-Analysis. Liver Cancer. Mar 2018;7(1):90-103.
45. Boshier PR, Heneghan R, Markar SR, Baracos VE, Low DE. Assessment of body

composition and sarcopenia in patients with esophageal cancer: a systematic review and meta-analysis. Dis Esophagus. Aug 1 2018;31(8).
46. Zhang G, Meng S, Li R, Ye J, Zhao L. Clinical significance of sarcopenia in the treatment of patients with primary hepatic malignancies, a systematic review and meta-analysis. Oncotarget. Nov 24 2017;8(60):102474-102485.
47. Shen Y, Hao Q, Zhou J, Dong B. The impact of frailty and sarcopenia on postoperative outcomes in older patients undergoing gastrectomy surgery: a systematic review and meta-analysis. BMC Geriatr. Aug 21 2017;17(1):188.
48. Sandini M, Pinotti E, Persico I, Picone D, Bellelli G, Gianotti L. Systematic review and meta-analysis of frailty as a predictor of morbidity and mortality after major abdominal surgery. BJS Open. Oct 2017;1(5):128-137.
49. Liu P, Hao Q, Hai S, Wang H, Cao L, Dong B. Sarcopenia as a predictor of all-cause mortality among community-dwelling older people: A systematic review and meta-analysis. Maturitas. Sep 2017;103:16-22.
50. Kim G, Kang SH, Kim MY, Baik SK. Prognostic value of sarcopenia in patients with liver cirrhosis: A systematic review and meta-analysis. Plos One. 2017;12(10):e0186990.
51. Jones K, Gordon-Weeks A, Coleman C, Silva M. Radiologically Determined Sarcopenia Predicts Morbidity and Mortality Following Abdominal Surgery: A Systematic Review and Meta-Analysis. World J Surg. Sep 2017;41(9):2266-2279.
52. Chang KV, Hsu TH, Wu WT, Huang KC, Han DS. Is sarcopenia associated with depression? A systematic review and meta-analysis of observational studies. Age Ageing. Sep 1 2017;46(5):738-746.
53. Shachar SS, Williams GR, Muss HB, Nishijima TF. Prognostic value of sarcopenia in adults with solid tumours: A meta-analysis and systematic review. Eur J Cancer. Apr 2016;57:58-67.
54. Chang KV, Hsu TH, Wu WT, Huang KC, Han DS. Association Between Sarcopenia and Cognitive Impairment: A Systematic Review and Meta-Analysis. J Am Med Dir Assoc. Dec 1 2016;17(12):1164 e1167-1164 e1115.
